# MoVam7, a Conserved SNARE Involved in Vacuole Assembly, Is Required for Growth, Endocytosis, ROS Accumulation, and Pathogenesis of *Magnaporthe oryzae*

**DOI:** 10.1371/journal.pone.0016439

**Published:** 2011-01-24

**Authors:** Xianying Dou, Qi Wang, Zhongqiang Qi, Wenwen Song, Wei Wang, Min Guo, Haifeng Zhang, Zhengguang Zhang, Ping Wang, Xiaobo Zheng

**Affiliations:** 1 Key Laboratory of Monitoring and Management of Crop Diseases and Pest Insects, Ministry of Agriculture, and Department of Plant Pathology, College of Plant Protection, Nanjing Agricultural University, Nanjing, China; 2 Department of Pediatrics and Research Institute for Children, Louisiana State University Health Sciences Center, New Orleans, Louisiana, United States of America; Institute of Developmental Biology and Cancer Research, France

## Abstract

Soluble NSF attachment protein receptor (SNARE) proteins play a central role in membrane fusion and vesicle transport of eukaryotic organisms including fungi. We previously identified MoSce22 as a homolog of *Saccharomyces cerevisiae* SNARE protein Sec22 to be involved in growth, stress resistance, and pathogenicity of *Magnaporthe oryzae.* Here, we provide evidences that MoVam7, an ortholog of *S. cerevisiae* SNARE protein Vam7, exerts conserved functions in vacuolar morphogenesis and functions in pathogenicity of *M. oryzae*. Staining with neutral red and FM4-64 revealed the presence of abnormal fragmented vacuoles and an absence of the Spitzenkörper body in the *ΔMovam7* mutant. The *ΔMovam7* mutant also exhibited reduced vegetative growth, poor conidiation, and failure to produce the infection structure appressorium. Additionally, treatments with cell wall perturbing agents indicated weakened cell walls and altered distributions of the cell wall component chitin. Furthermore, the *ΔMovam7* mutant showed a reduced accumulation of reactive oxygen species (ROS) in the hyphal apex and failed to cause diseases on the rice plant. In summary, our studies indicate that MoVam7, like MoSec22, is a component of the SNARE complex whose functions in vacuole assembly also underlies the growth, conidiation, appressorium formation, and pathogenicity of *M. oryzae*. Further studies of MoVam7, MoSec22, and additional members of the SNARE complex are likely to reveal critical mechanisms in vacuole formation and membrane trafficking that is linked to fungal pathogenicity.

## Introduction

Exocytosis and endocytosis are essential for fungal growth, and virulence. Like in other eukaryotic organisms the secretory processes in fungi require many steps of vesicular traffic between distinct membrane-bound organelles. Secretory materials include extracellular enzymes and others leave the endoplasmic reticulum (ER) in vesicles to Golgi apparatus (GA). Following passage through the GA the cargos are packaged into vesicles destined for the plasma membrane (PM). These vesicles either fuse to the PM or exocytose their contents. Complementary to the secretory pathway, there is an endocytic pathway that internalizes extracellular materials and retrieves membrane and proteins from the PM. In the course of endocytosis, portions of the PM invaginate into pits and pinch off as vesicles into the cytoplasm. Some of the internalized material is delivered to the degradative vacuole after passage through early and late endosomes. The remainder may leave the endosomes and reenter the secretory route indirectly via the GA or directly via recycling to the PM. Several studies support the existence of endocytosis in filamentous fungi, with FM4-64, a specific tracer of endocytosis is incorporated into the Spitzenkörper, suggesting this organelle may be involved in endocytic membrane recycling [1], [2], [3], [4], [5], [6].

SNARE proteins are a family of conserved proteins involved in the intracellular transport of membrane-coated cargos from one sub-cellular compartment to another. Despite their different sizes and structures among many organisms, the SNARE proteins share a conserved structure called the SNARE motif, which consists of 60–70 amino acids arranged in heptad repeats [7], [8]. Based on its sequence similarity, the SNARE superfamily is further divided into four subfamilies: Qa, Qb, Qc, and R [9]. SNAREs seem to mediate membrane fusion in all of the trafficking steps of the secretory pathway. According to the current molecular model of SNARE-mediated membrane fusion, SNARE proteins localized in opposing membranes drive membrane fusion by using the free energy that is released during the formation of a four helix bundle. The formation of this bundle leads to a tight connection of the membranes that are destined to fuse, and initiates the membrane merger. The recycling of SNAREs is achieved through the dissociation of the helical bundle, which is mediated by the ATPases associated with diverse cellular activities (AAA+) protein NSF (N-ethylmaleimide-sensitive factor) [10]. SNAREs have been extensively studied in mammals and in the budding yeast *Saccharomyces cerevisiae*, investigations of their roles in filamentous fungi, particularly those that are capable of causing crop diseases, remain very limited [3], [11], [12]. With the availability of many genome sequences, SNARE proteins were also identified from various filamentous fungi. Kuratsu and colleagues identified 21 putative SNARE proteins from *Aspergillus oryzae*, and they also determined the localization of these putative SNAREs using eGFP marker fused chimeras constructs [13], [14].

The rice blast fungus *Magnaporthe oryzae* was widely regarded as a model fungus because of its economic and social significance, as well as its amenability to genetic and other means of analysis [15], [16], [17]. We have previously characterized MoSec22, a member of *M. oryzae* SNARE complex and found that it has a role not only in membrane trafficking but also in growth, stress tolerance, and pathogenicity [18]. As a part of our continuing research efforts to examine the roles of membrane trafficking in fungal pathogenesis, we characterized the function of MoVam7 in *M. oryzae*, whose yeast homolog is a conserved component of the SNARE complex regulating vacuolar assembly and membrane fusion. We found that MoVam7 exhibits distinct as well as redundant functions with MoSec22 in intracellular transport affecting growth, differentiation, and virulence of the fungus.

## Materials and Methods

### Fungal strains and medium

*M. oryzae* strain Guy11 was used as the wild type strain and all strains were cultured at 28°C on complete CM medium [19]. Other media were minimal medium (MM: 6 g NaNO_3_, 0.52 g KCl, 0.52 g MgSO_4_, 1.52 g KH_2_PO_4_, 10 g glucose, and 0.5% biotin in 1 L distilled water), OMA medium (30 g oat meal and 10 g agar in 1 L distilled water), and RDC (100 g of rice straw decoction to 1 L double-distilled (dd) H_2_O, boiled for 20 min and filtered. 40 g cornmeal and 10 g agar were added and volume adjusted to 1 L with distilled water) [20], [21]. Mycelia were harvested from 3-day old culture in liquid CM by filtration and used for genomic DNA and total RNA extractions.

### Cloning and sequence analysis of the *MoVAM7* gene

A full-length cDNA fragment for the *MoVAM7* gene was isolated from Guy11 strain using primers FL1466/FL1467. cDNA was cloned into pMD19 T-vector (TaKaRa, Dalian, China) to generate pMD-*MoVAM7* and verified by sequencing. Amino acid sequence alignments were performed using the Clustal_W program [22] and the calculated phylogenetic tree was viewed using Mega 3.1 Beta program [23].

### Disruption of *MoVAM7* and Δ*Movam7* mutant complementation

The vector pMD-*MoVAM7*KO was constructed for targeted gene deletion by inserting the *HPH* marker gene cassette into the two flanking sequences of the *MoVAM7* gene. A 1.0 kb upstream flanking sequence and a 0.8 kb downstream flanking sequence were amplified with primer pairs FL724/FL725 and FL726/FL727, respectively. Two PCR fragments were linked by overlap PCR with primer pairs FL724/FL727, and the linked sequence cloned into pMD19 T-vector. The *HPH* gene cassette, amplified with primers FL1111/FL1112, was inserted into pMD-*MoVAM7* at the *Eco*RV site (incorporated into primers FL725 and FL726) to generate the final pMD-*MoVAM7*KO. A 3.2 kb fragment containing the *MoVAM7* disruption allele was amplified using primers FL724/FL727, purified, and used to transform the Guy11 strain according to established protocol [19]. Table S1 lists all of the primers used.

Putative Δ*Movam7* mutants were screened by PCR and confirmed by Southern blotting analysis. Further confirmation was in the form of transcript detection through use of RT-PCR with primers FL2194/FL2195 (Figure S1). For complementation, a 4 kb PCR product containing the full-length *MoVAM7* coding region, as well as 2 kb upstream and 0.5 kb downstream sequences, was amplified using primers FL1464/FL1465 and subcloned into pCB1532 generating pCB1532-*MoVAM7*R. The resulting transformants were first screened by phenotype characterization followed by PCR amplification.

### PCR amplification

Total RNA was isolated using the NucleoSpin RNAII RNA extraction kit (Macherey-Nagel, Bethlehem, PA). First-strand cDNA was synthesized using the M-MLV Reverse Transcriptase (Invitrogen Co., Shanghai, China) and oligo(dT) 15 Primers (TaKaRa Co., Dalian, China) synthesis system. For semi-quantitative RT-PCR, a 0.3 kb fragment for the actin gene (MGG_03982.5) was amplified using primers FL474 and FL475 and used as an internal control (Table S1).

Quantitative RT-PCR was performed using the ABI 7300 Real-Time PCR System (Applied Biosystems, Foster City, CA) following the manufacturer's instructions. Transcription of chitin synthases genes MGG_01802 (*CHS2*), MGG_04145 (*CHS1*), MGG_09551 (*CHS3*), MGG_06064 (*CHS7*), MGG_09962 (*CHS4*), MGG_13013 (*CSM1*), and MGG_13014 (*CHS6*) were analyzed using primer pairs FL4929/FL4930, FL4931/FL4932, FL4933/FL4934, FL4935/FL4936, FL4937/FL4938, FL4939/FL4940, and FL4941/FL4942, respectively. Transcription of 9 putative genes encoding laccase with signal peptide were analyzed using specific primer pairs. The actin gene, amplified with primers FL4362 and FL4363, was used as an internal control. The above primers used in this section were listed in Table S1.

### Dry weight determination and protoplast release assay

Equal plugs of mycelia, from 7-day-old CM plates were transferred into liquid CM or V8. The mycelia were cultured with shaking (125 rpm) at 25°C for 3 days. Fungal biomass was recorded by dry weight determination of three replicates per treatment in three independent experiments.

Mycelia were cultured in CM liquid medium for 2 days and harvested by centrifugation for 10 min at 5,000× g. The mycelia were washed twice and resuspended with 20% sucrose. Lysing enzyme (Sigma-Aldrich, MO, USA) was added to the suspension (2 mg ml^–1^), with lysis stopped after 30, and 90 min of incubation by placing the reaction tubes in ice. The protoplasts were separated from the mycelia by filtration through Miracloth. Protoplast release and cell wall degradation were examined with a light microscope and counted using a hemacytometer.

### Assays for vegetative growth, conidiation and appressorium formation

For growth, mycelia plugs of 3 mm ×3 mm were transferred from 7-day-old CM plates and inoculated on fresh medium (CM, MM, V8, OMA, and RTC) followed by incubation at 28°C with a 12 hour interval photophase. The radial growth was measured after incubation for 6 days. All the experiments were repeated three times each with three replicates.

For conidia production, mycelia were grown in the dark on RDC medium at 28°C for 7 days followed by constant illumination for 3–4 days. Conidia were collected by washing with ddH_2_O, filtered through three-layer lens paper, and concentrated by centrifugation (6,000×g) at 4°C for 10 min. The final spores were suspended into 0.2 ml ddH_2_O and counted using a hemacytometer.

Appressorium formation was measured on GelBond film (FMC Bioproducts, Rockland, Maine, USA) as previously described [24]. Appressoria were observed through direct microscopic examination and percentages obtained from at least 100 pieces of fragmented mycelia per replicate at 24 and 48 hours in at least three experiments.

### Appressorium formation, invasive hyphal growth and pathogenicity test

For pathogenicity assessment, mycelia cultured in CM liquid medium for 2 days were harvested (5,000×g, 2 min), washed twice with distilled water, and fragmented into the length of 30–50 µm using a homogenizer. The mycelia suspension was adjusted to 5×10^4^ pieces/ml and was inoculated onto intact or abraded leaves of the susceptible rice variety *Oryzae sativa* cv CO39. Appressoria penetration and infectious hyphal growth were monitored daily and photographed under a phase-contrast microscope.

### Light and transmission electron microscopy

To study the hyphal morphology, strains were grown on microscope slides carrying a thin layer of CM agar and observed under an Olympus BH-2 microscope (Olympus, Japan). Calcofluor White (CFW) staining was performed as described [25]. FM4-64 and neutral red staining were conducted following procedures previously described [2], [26]. Photographs were taken under the Leica DMR microscope (Leica Microsystems, Wetzlar, Germany). The Spitzenkörper was observed and photographed under a Leica TCS SP2 confocal microscope (Leica, Germany).

For transmission electron microscopy, mycelia mass was collected, fixed for 1 hour at 4°C in 50 mM sodium phosphate buffer (pH 7.2) containing 3% glutaraldehyde and 2% paraformaldehyde, and washed three times, 10 min each time, with 0.1 M phosphate buffer (pH 7.2). The samples were post-fixed in 1% OsO_4_ for 2 hours, washed three times with PBS, and dehydrated in a graded ethanol series. The sample was then embedded in Spurr resin and stained with 2% uranyl acetate and Reynold's lead solution before sectioning. The ultrathin sections were examined under a JEM-1230 electron microscope operating at 80 kV (Hitachi-7650, Tokyo, Japan).

### Hydrogen peroxide (H_2_O_2_) determination, reaction oxygen species (ROS) detection, and measurement of the extracellular laccase activity

Mycelia were cultured in CM liquid medium for 2 days and harvested by centrifugation for 10 min at 5,000×g. The level of H_2_O_2_ was analyzed by a commercially available Kit (Beyotime Institute of Biotechnology, China) and measured the absorbance at 560 nm using a microplate reader (BioRad-Benchmark, USA).

Intracellular ROS levels were monitored using the oxidant-sensitive probe dihydrorhodamine-123 (Molecular Probes, Carlsbad, CA). *M. oryzae* strains grown on microscope slides overlaid with CM agar for 2 days were incubated with 50 mM dihydrorhodamine-123 for 2 hours at room temperature, rinsed twice with PBS, and then observed under the microscope [21].

For superoxide detection, the hyphae of the wild-type strain Guy11 and *ΔMovam7* mutants were collected from 3-day-old CM agar plates and stained with 0.6 mM nitroblue tetrazolium (NBT) aqueous solution for 2 hours after which the reaction was stopped by adding ethanol. Hyphal tips were viewed under a bright-field microscope for the production of the superoxide [27].

The laccase activity was monitored on 0.2 mM 2, 2′-azino-di-3-ethylbenzath- iazoline-6-sulfonate (ABTS) agar plate assays using mycelial plugs at 2 days post infection. The enzyme activity was also assayed using the culture filtrate from 3 day old CM liquid culture. Briefly, a reaction mixture (1 ml) containing 50 mM acetate buffer (pH 5.0) and 10 mM ABTS was mixed with the culture filtrate (200 ml) and incubated at 25°C for 5 minutes. Absorbance was evaluated at 420 nm [18], [27].

### Chitin content assessment

Chitin (N-acetylglucosamine, GlcNAc) content was determined as described [18]. Mycelial samples were freeze-dried first. For each sample, 5 mg of dried biomass was resuspended in 1 ml 6% KOH and heated at 80°C for 90 min. Samples were centrifuged (16,000×g, 10 min) and pellets washed with PBS in three cycles of centrifugation and resuspension (16,000×g, 10 min). The pellets were finally resuspended in 0.5 ml of McIlvaine's buffer (pH 6) with 100 ml (13 units) of *Streptomyces plicatus* chitinase (Sigma, USA) and incubated for 16 hours at 37°C with gentle mixing. 100 ml sample was then combined with 100 ml of 0.27 M Mosadium borate (pH 9) in a 1.5 ml Eppendorf tube, heated for 10 min at 100°C, and 1 ml of freshly diluted (1∶10) of Ehrlich's reagent (10 g β-dimethylaminobenzaldehyde in 1.25 ml of concentrated HCl and 8.75 ml glacial acetic acid) was added. After incubating at 37°C for 20 min, 1 ml of the sample was transferred to a 2.5 ml plastic cuvette (Greiner) and the absorbance at 585 nm was recorded. Standard curves were prepared with GlcNAc (Sigma, USA). The experiment was repeated three times.

## Results

### *M. oryzae* MoVam7 is a paralog of *S. cerevisiae* Vam7

**Figure 1:**
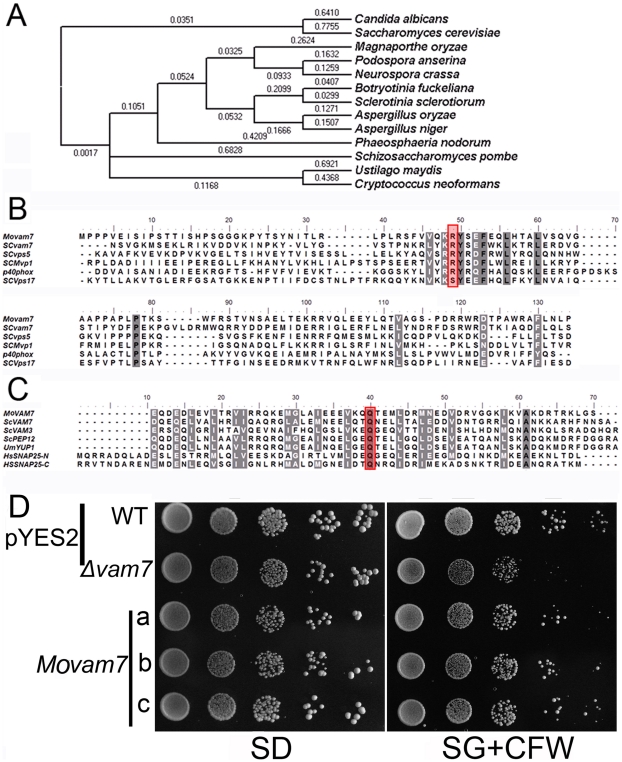
*Magnaporthe oryzae* Vam7 shares sequence homology with several other fungal Vam7 proteins. (A) Dendrogram of various Vam7 homologs in fungi: MoVam7 (*Magnaporthe oryzae*), XP_001907727.1 (*Podospora anserine*), XP_001551407.1 (*Botryotinia fuckeliana*), XP_001591719.1 (*Sclerotinia sclerotiorum*), XP_957713.1 (*Neurospora crassa*), BAF36378.1 (*Aspergillus oryzae*), XP_001391677.1 (*Aspergillus niger*), XP_001804975.1 (*Phaeosphaeria nodorum*), XP_567839.1 (*Cryptococcus neoformans*), XP_712444.1 (*Candida albicans*), NP_011303.1 (*Saccharomyces cerevisiae*), and XP_761553.1 (*Ustilago maydis*). Sequence alignments were performed using the Clustal_W program and the calculated phylogenetic tree was viewed using Mega3.1 Beta program Neighbor-joining tree with 1000 bootstrap replicates of phylogenetic relationships between VAM7 homologs in fungi. Numbers above the branches represent amino acid substitution. (B) Similarity of the MoVam7 PX domain to those that specifically bind ptdIns(3)P. The amino acid alignment of the PX domains for *M. oryzae* (MoVam7), *S. cerevisiae* (ScVam7, NP_011303.1), *S. cerevisiae* (ScVps5, EDV10670.1), *S. cerevisiae* (ScMvp1, EDV11508.1), *S. cerevisiae* (ScVps17, NP_014775.1), and humans (Hs p40phox, NP_000622.2) was performed using Clustal_W [22]. Positions corresponding to Arg58 were shaded. (C) Alignment of the SNARE motifs suggesting that MoVam7 is a t-SNARE protein. SNARE motifs origins: ScVam7 (NP_011303.1), ScVam3 (EDN63963), UmYup1 (XP761553), Scpep12 (NP014624), and HsSnap25 (NP570824). (D) The *MoVAM7* gene rescued the CFW sensitivity of *S. cerevisiae* Δ*vam7* mutant. The yeast Δ*vam7* mutant was transformed with the pYES2–*MoVAM7* construct expressing MoVam7. The wild type strain was also transformed with the empty pYES2 vector as a control. Serial dilutions of cultures of three independent transformants (a, b, and c) were grown overnight on SD-Met-Leu-His (glucose) or SG-Met-Leu-His (galactose +125 µg/ml CFW) plates, and grown at 30°C for 4 days and photographed. The experiment was repeated at least three times and representative results were photographed.

Examination of the *M. oryzae* genome database at the Broad Institute (www.broad.mit.edu/annotation/genome/magnaporthe_grisea/Home.html) revealed that MGG_05428.5 shares high amino acid sequence homology to several fungal SNARE proteins, including *S. cerevisiae* Vam7p (Figure 1A). We named MGG_05428.5 as MoVam7, which appears to be encoded by a single copy gene (Figure S1).

With an overall 20% amino acid sequence identity, MoVam7 is also considerably divergent from *S. cerevisiae* Vam7 with the exception of the two domains that were quite conserved. The N-terminus of MoVam7 is predicted to have a PHOX homology domain (PX), which was originally identified as a common motif of about 120 amino acids found in the P40phox and P47phox subunits of the neutrophil NADPH oxidase (PHOX) [28]. It was also found that in the PX domains that specifically bind to phosphatidylinositol-3-phosphate [ptdIns(3)P], the amino acid residue corresponding to Arg58 is a basic amino acid, whereas those lacking the analogous basic residue exhibited alternative binding specificity for phosphoinositides [29], [30], [31]. The PX domain of MoVam7 has an Arg residue in the corresponding position, indicating that MoVam7 preferably binds ptdIns(3)P (Figure 1B). The C terminus of MoVam7 contains a SNARE motif arranged in heptad repeats, which is predicted to encode a v-SNARE, but further examination of the C-terminal SNARE motif revealed that the central position (0-layer) of the heptad repeats is a Gln (Q) residue, which is conserved in all t-SNARE proteins, including Vam7 of *S. cerevisiae* and Yup1 of *U. maydis* (Figure 1C).

In *S. cerevisiae*, the *Δvam7* mutant is sensitive to CFW [32], and also exhibited fragmented vacuoles due to a defect in homeotypic vacuolar fusion, instead of a large vacuole compartment [33]. To determine if MoVam7 can substitute for the functions of *S. cerevisiae* Vam7, the yeast Δ*vam7* mutant was transformed with pYES2 containing the *MoVAM7* gene. The resulting transformant expressing *MoVAM7* showed the restored growth on medium containing 125 µg/ml CFW, in comparison to the strain transformed with the pYES2 vector alone (Figure 1D). These results indicated that MoVam7 is a functional paralog of *S. cerevisiae* Vam7.

### *Movam7* deletion affects endocytosis and formation of the Spitzenkörper

**Figure 2:**
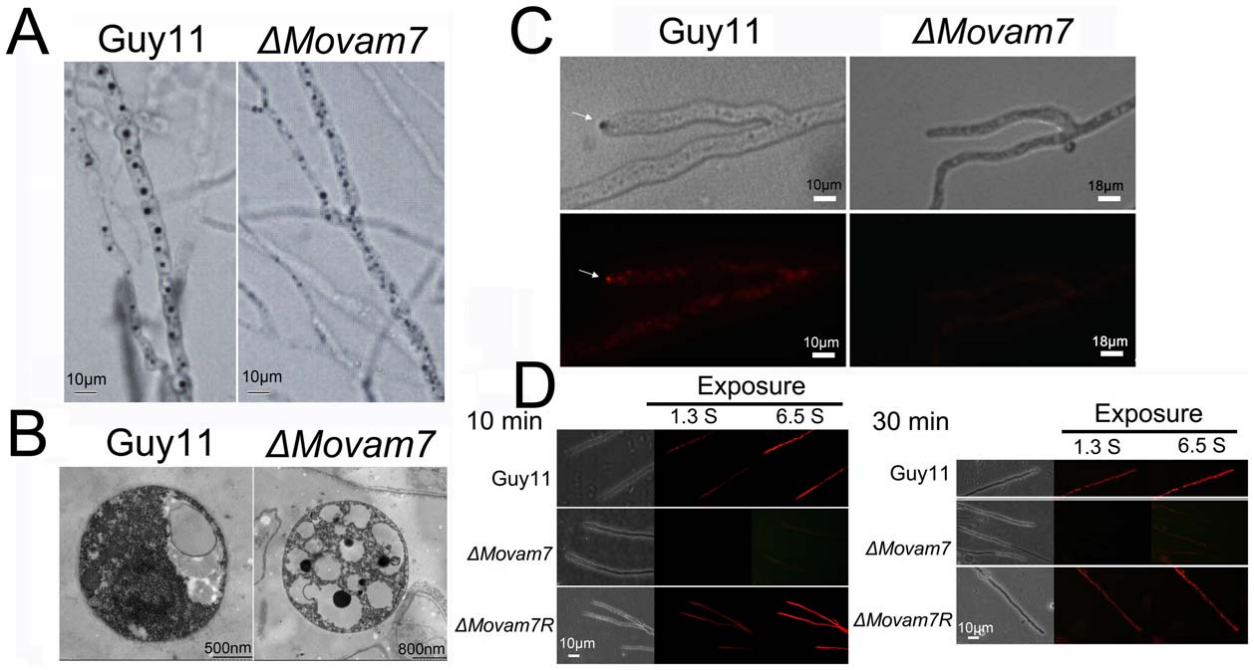
MoVam7 has a role in vacuole morphogenesis and the formation of the Spitzenkörper and endocytosis. (A) Mycelia were stained with neutral red. At 14 hours, numerous small vacuoles were seen in the Δ*Movam7* mutants (right panel), whereas large vacuoles of fewer numbers were present in the wild type strain. (B) Observation by transmission electron microscopy revealed numerous small, fragmented vacuoles in contrast to the large ones in the wild type hyphae. Bars represent 500 nm in the wild type strain or 800 nm in the Δ*Movam7* mutant. (C) The wild type strain shows the presence of an intact Spitzenkörper (arrowheads) at the tips of the hyphae, which was missing in the Δ*Movam7* mutants after exposure to FM4-64 staining for 10 min. Strains were grown for 2 days on CM-overlaid microscope slides before staining. (D) FM4-64 staining also revealed that the Δ*Movam7* mutant was defective in endocytosis. Strains were grown for 2 days on the CM-overlaid microscope slides before adding FM4-64 and photographs were taken after 10 and 30 min exposure to FM4-64. Camera exposure is indicated in second.

As MoVam7 was identified based on its homology to *S. cerevisiae* Vam7, which is a SNARE protein involved in vacuole assembly and membrane fusion, we performed staining with vital dyes such as neutral red and FM4-64. In normal cells, neutral red is quickly internalized allowing staining of vesicles such as vacuoles. The Δ*Movam7* mutants displayed vacuole formation by neutral red staining and transmission electron microscopy (Figure 2A and 2B). However, the vacuoles were smaller and more numerous (Figure 2A and 2B), which was similar to the observed phenotypes of the Δ*vam7* mutant of *S. cerevisiae* [34], [35], indicating a role for MoVam7 in vacuolar formation/assembly.

The Spitzenkörper is a complex, multicomponent structure dominated by vesicles. Recent studies suggested Spitzenkörper is involved in both exocytosis and endocytosis [36], [37]. We examined the formation of the Spitzenkörper with FM4-64 staining. The Spitzenkörper was readily seen in the wild-type strain but not in the Δ*Movam7* mutants (Figure 2C). To explore whether the loss of MoVam7 resulted in an endocytosis defect, we followed the uptake of FM4-64 over a time course. In the wild-type strain, FM4-64 appeared in the plasma membrane within 5 min and in the endomembrane consisting of vacuoles and endosomes within 10 min (Figure 2D). In contrast, dye uptake in the Δ*Movam7* mutants was not apparent at 5 min. At 30 min, the dye remained on the plasma membrane, and weak fluorescence was seen internally at 90 min, which was likely due to diffusion (Figur 2D). These results suggested that disruption of *MoVAM7* resulted in endocytosis defect and affected the proper maintenance of Spitzenkörper morphology.

### MoVam7 affects vegetative growth and sporulation

**Figure 3:**
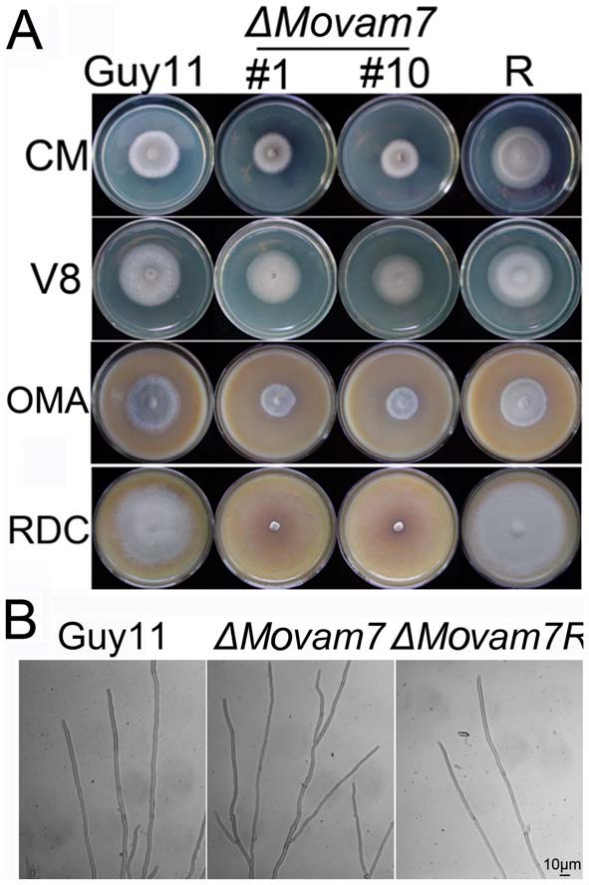
MoVam7 is required for normal growth and hyphal morphology. (A) The Δ*Movam7* mutants displayed retarded vegetative growth on CM, V8 and OMA medium, and the mutants also produced few aerial hyphae on RDC medium in comparison to the wild-type strain Guy11. This experiment was repeated three times and representative colonies were photographed. (B) The hyphae of the Δ*Movam7* mutants are highly branched and curled. The Δ*Movam7*, reconstituted (Δ*Movam7R*), and wild-type strains were cultured on the CM-overlaid microscope slides for 2 days and the mycelia were microscopically observed. Representative colonies were photographed. Bars equal 10 µm.

We evaluated the growth of Δ*Movam7* mutants on CM, V8, oatmeal, and RDC media plates. The Δ*Movam7* mutants displayed reduced growth on CM in comparison to the wild-type Guy11 and complemented Δ*Movam7R* strains. The mutant's colony also appeared thinner and lacked aerial hyphae. On RDC medium, Δ*Movam7* mutants formed a thin white colony that gradually collapsed into a film-like material (Figure 3A). Additionally, the hyphae of the mutant appeared more branched (Figure 3B). To further assess the effect of MoVam7 on growth, the dry weight of cultures grown on CM and V8 media was determined. The Δ*Movam7* mutants showed 15% reduction in dry weight on CM medium, but 23% reduction on was found on V8 medium than the wild-type strain (Figure S2).

**Figure 4:**
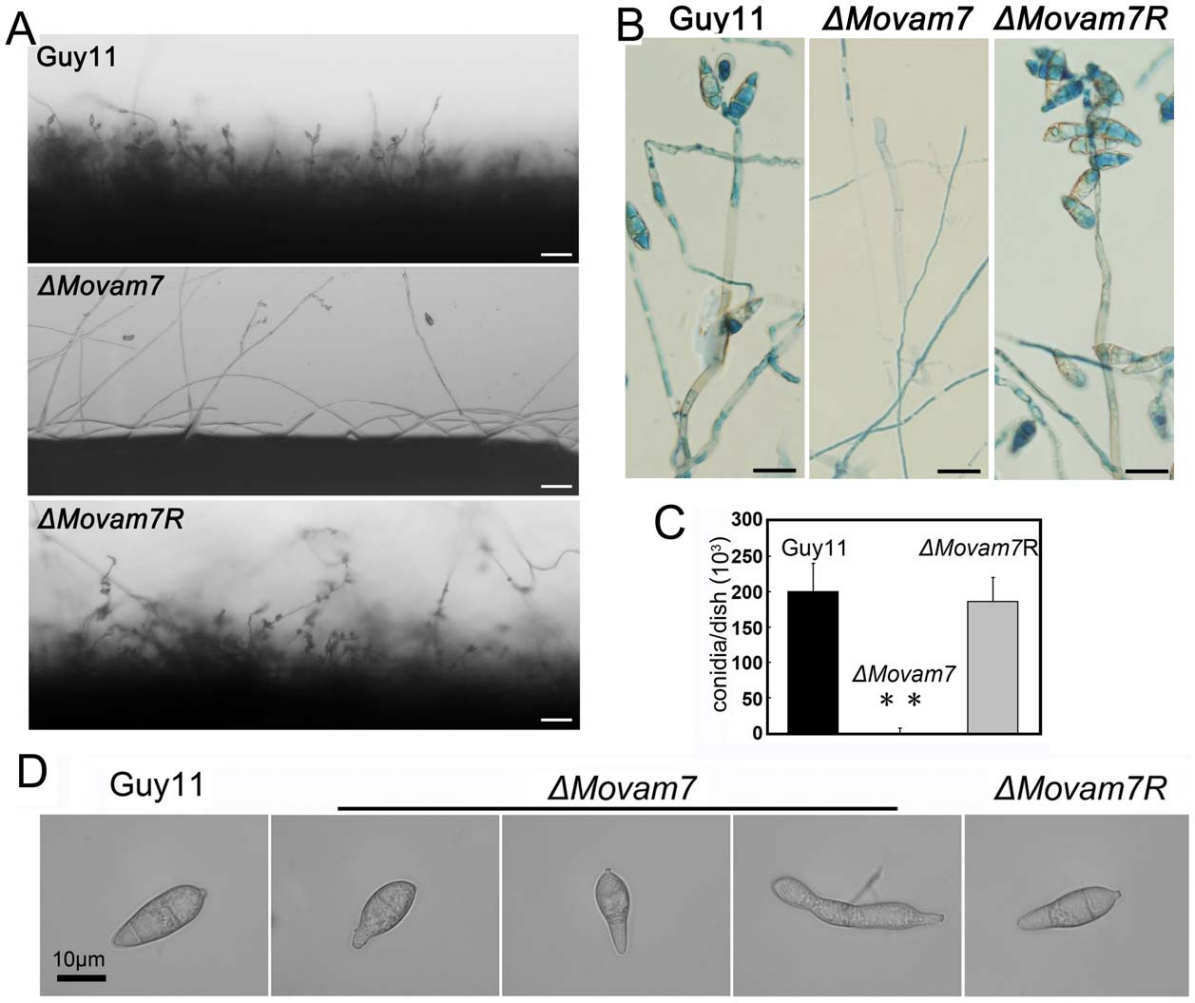
MoVam7 is required for normal conidia formation. (A) Development conidia on conidiophores. Light microscopic observation was performed on strains grown on RDC medium for 9 days. Bars  = 100 µm. (B) Aerial structures stained with lactophenol aniline blue. Conidia and aerial hyphae stained blue, and conidiophores stained gray. Bars  = 30 µm. (C) Few conidia were found in the Δ*Movam7* mutants in contrast to the wild type and complemented Δ*Movam7* strains. Panel indicates conidia counts per plate. Error bars represent the standard deviation and asterisks represent significant different among stains tested. (D) Rare conidia of the Δ*Movam7* mutant displayed abnormal morphology with no or only one septa, whereas the normal oblong shaped spores with tapered ends and multiple septa were present in the wild type and complemented Δ*Movam7R* strains.

We also examined the effect of *MoVAM7* disruption on the production of conidia. We found that the Δ*Movam7* mutants produced no spores on CM, OMA, and V8 medium, and only small amounts of irregular conidia were found in the pre-autolysis scrapings off RDC medium (Figure 4A, 4B and 4C). In these spores, there were either no or one septa, instead of two septa as seen in the wild type or complemented strains (Figure 4D). We further analyzed the conidiophore differentiation and conidial formation in the *ΔMoavm7* mutant. As shown in Figure 4A, small amounts of conidiophores were observed in the mutant at 24 hours post conidial induction after 8-day old incubation. In order to determine if MoVam7 is involved in conidiophore development, staining with lactophenol aniline blue was used to distinguish conidiophores from other aerial hyphae [18], [38]. Microscopic examination revealed that conidiophores developed in the Δ*Movam7* mutant, as in the wild type and Δ*Movam7R* strains (Figure 4B). These data suggested that MoVam7 is required for conidial formation.

### Deletion of Movam7 alters chitin distribution

**Figure 5:**
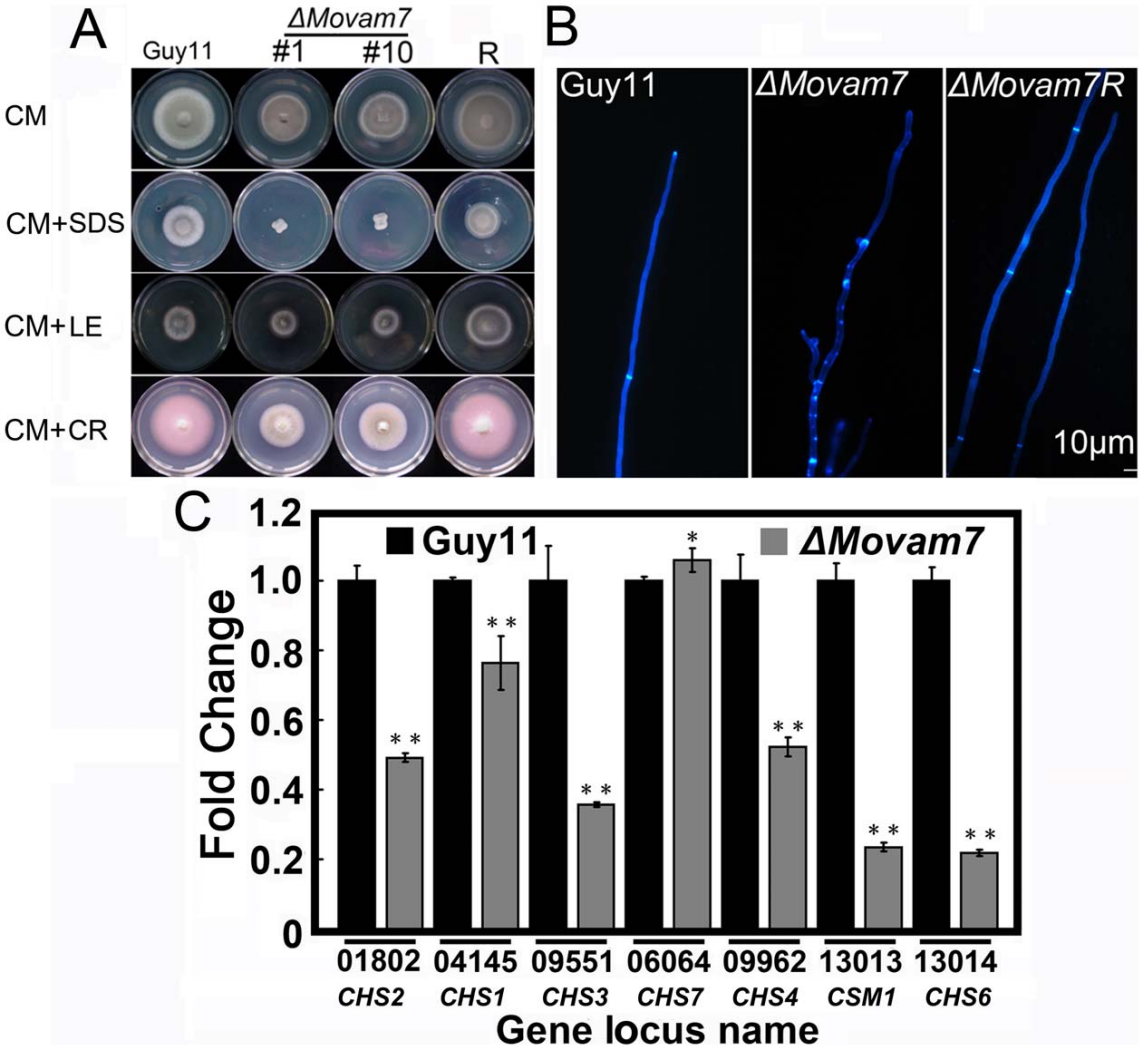
Deletion of *Movam7* resulted in altered distribution of chitins. (A) The Δ*Movam7* mutants (#1 and #10), complemented mutant (Δ*Movam7R*), and wild type strains were incubated on CM plates supplemented with various stress inducers at 28°C for 6 days. Growth of the Δ*Movam7* mutant in media supplemented with SDS (0.01%; vs wild type *P*<0.01), lysing enzymes (2 mg/ml), and Congo Red (20 µg/ml). (B) Deletion of *Movam7* altered the distribution of chitin within the cell. In the wild-type strain Guy11, CFW fluorescence was mainly distributed at the hyphal and septal apices, whereas in the Δ*Movam7* mutant, fluorescence was not restricted to growing apices and found also on the lateral walls along the hyphal axe. This abnormal distribution of cell wall components was restored by the introduction of the wild-type *MoVAM7* gene. (C) Reduced expression was found in six out of seven genes that encode chitin synthases in the Δ*Movam7* mutants of *M. oryzae*. RNA was extracted from mycelia that were grown for 3 days in liquid CM. Error bars represent the standard deviation and asterisks represent significant different among stains tested. All of the reductions are significant (*P* = 0.01 or *P* = 0.05) according to Duncan's multiple range test.

To examine the role of MoVam7 in cell wall integrity, we investigated the effects of various cell-wall perturbing agents on the Δ*Movam7* mutant. The sensitivity of the Δ*Movam7* mutant to lytic enzymes (LE), and Congo Red (CR) is not significantly different from that of the wild–type strain and the complemented mutant strains, but it was more sensitive to SDS (Figure 5A). No significant difference in the rate of protoplast release was found at 30 min and 90 min (Figure S3). CFW staining was also used to probe the distribution of chitin on the cell wall. In the wild type strain, CFW fluorescence was mostly distributed at the septa and tips where chitin, one of main components of the fungal cell wall, was actively synthesized, while in the mutants, patches of bright fluorescence were observed on the lateral wall of hyphae in addition to septa and hyphal tips (Figure 5B). Since CFW is a fluorescent dye that intercalates with nascent chitin chains, this result might suggest the altered distribution of chitin on cell wall in the Δ*Movam7* mutant was due to the aberrant cell wall synthesis activity. Chitin contents analysis showed the chitin contents were reduced by 10% in the Δ*Movam7* mutant compared to the wild-type strain (Figure S4). We also examined the expression of several genes encoding the putative chitin synthases in *M. oryzae* by quantitative RT-PCR assay. Interestingly, the result showed that with the exception of MGG_06064, the expression for six out of seven genes was reduced in the Δ*Movam7* mutant in comparison to the wild strain (Figure 5C). These findings indicated that the aberrant chitin distribution, combining with reduced chitin production due to reduced gene expression, contributed together to weakened altered cell wall integrity in Δ*Movam7* mutants.

### MoVam7 affects the accumulation of ROS *in vivo*

**Figure 6:**
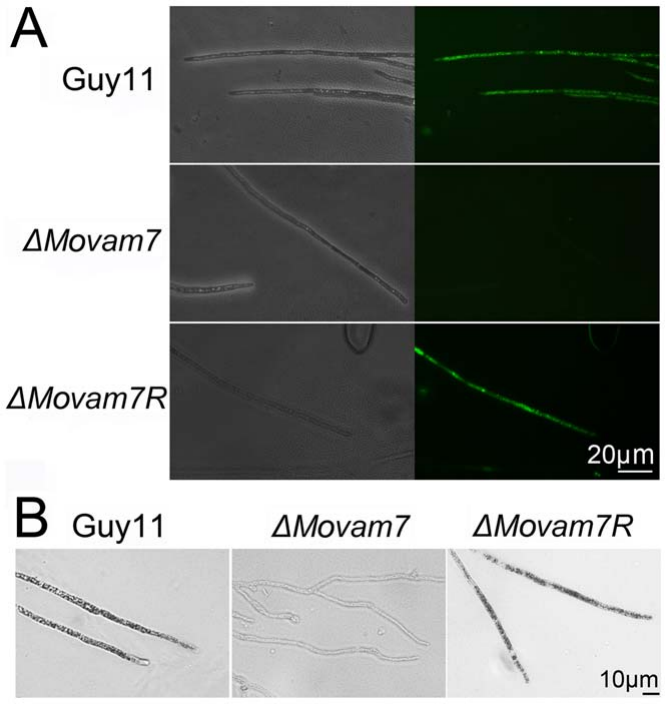
MoVam7 is required for *in vivo* ROS accumulation. (A) In the wild-type strain Guy11, intense fluorescence suggested a high ROS level *in vivo*. The tips of Δ*Movam7* mutants displayed weak fluorescence at the same exposure time, suggesting that the disruption of the *Movam7* gene reduced ROS accumulation. The tips of the reconstituted strain showed bright fluorescence similar to the wild type strain. All strains were grown for 2 days at room temperature on CM-overlaid microscope slides before staining. The experiments were repeated multiple times each with similar results. (B) Dark NBT stain in the wild-type strain also suggests a high ROS level *in vivo*, in contrast to the Δ*Movam7* mutant that displayed barely visible stain on the tips of the hyphae, suggesting the reduced ROS accumulation. The tips of the complemented Δ*Movam7R* strains recovered the deep color, as expected. All of the strains were grown for 2 days at room temperature on CM-overlaid microscope slides before staining. The experiments were repeated multiple times.

The production of ROS is considered to be critical for the development of infection related morphogenesis in *M. oryzae* [39], and ROS presence at the metabolically active hyphal tips can be visualized using dihydrorhodamine-123. We found that the fluorescence emission at the hyphal tips was significantly less apparent in the Δ*Movam7* mutant than in the wild-type Guy11 and complemented strains (Figure 6A), indicating a reduced ROS accumulation. We also analyzed the content of the superoxide using NBT staining. The Δ*Movam7* mutant contained the lower superoxide content (less pigmented), consistent with the finding employing dihydrorhodamine-123 (Figure 6B). Furthermore we determined the level of H_2_O_2_, the dismutation product of superoxide in the Δ*Movam7* mutant and found that the level of H_2_O_2_ was significantly reduced in the Δ*Movam7* mutants (Figure S5). These results indicated that loss of MoVam7 function affects the accumulation of ROS, especially the accumulation of the superoxide.

### MoVam7 is involved in the secretory transport of extracellular laccases

**Figure 7:**
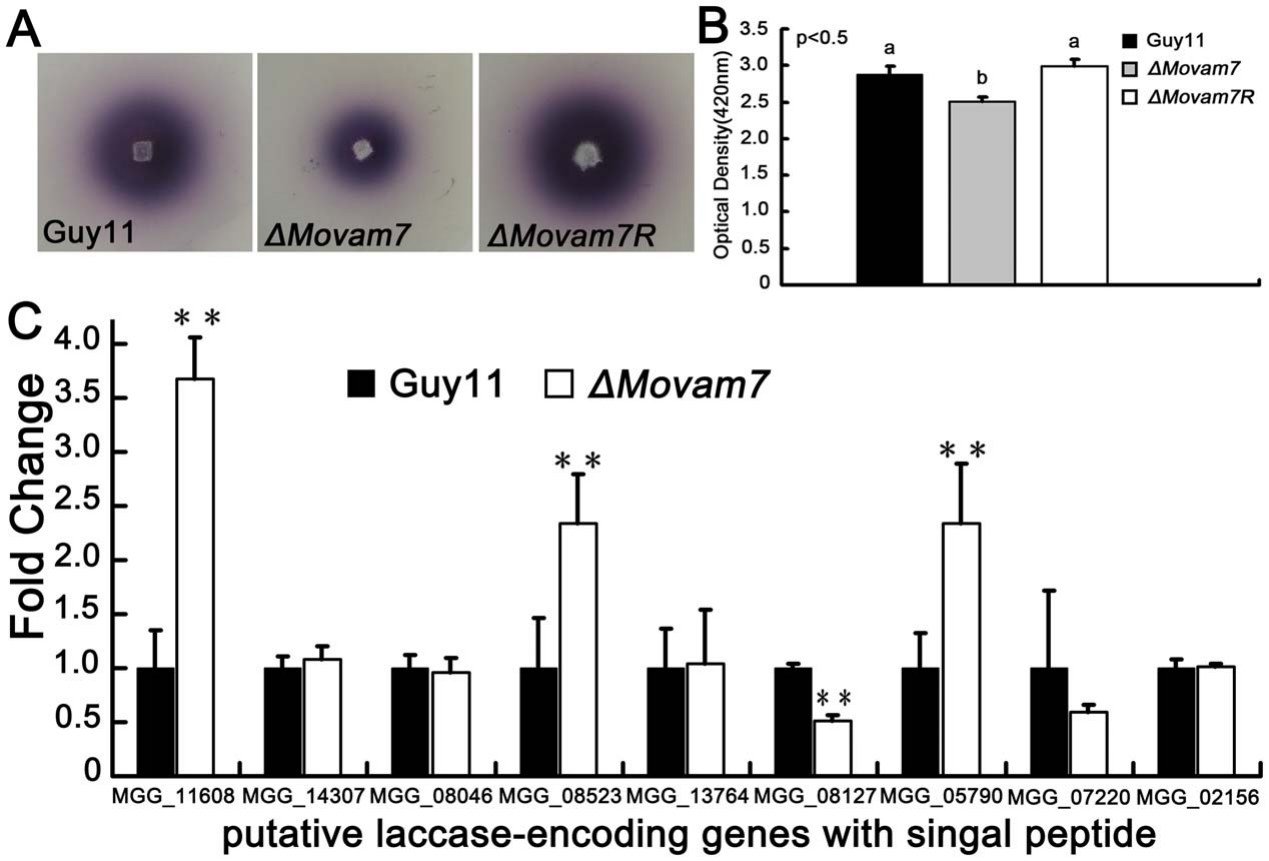
MoVam7 regulates laccase secretion. (A) The laccase activity was monitored in complete media supplemented with 0.2 mM ABTS 3 days after inoculation. Three independent experiments with triplicate replicates were performed. (B) The laccase were measured by the ABTS oxidization test. In all experiments, three independent experiments were carried out with triplicate replicates each time. Error bars represent the standard deviations where applicable. (C) No significantly reduced expression was found in eight of 9 putative genes encoding laccase with signal peptide. RNA extraction, PCR reaction, and statistical analysis were the same as in Figure 5C.

A previous report suggested that a Vam7-like SNARE protein of *U. maydis*, Yup1, could couple endocytosis and exocytosis by assisting membrane recycling processes [3]. As mentioned above, the Δ*Movam7* mutant displayed aberrant chitin distribution on the cell wall, which is similar to the phenotype of Δ*yup1* mutant of *U. maydis*. Aberrant chitin distribution could result from a defect in secretory transport of chitin-containing chitosomes. Laccase is also one of the secretory extracellular proteins that can be easily assessed. The laccase activity was measured from cells grown on solid medium and in the culture filtrate, which showed that the Δ*Movam7* mutant has a moderate reduction in the laccase activity (Figure 7A and 7B). To determine laccase expression levels, 9 putative genes encoding laccases with signal peptide were selected from the *M. oryzae* genome database for qRT-PCR. Transcripts of five of the 9 genes were unchanged in the Δ*Movam7* mutant. Only one of nine genes encoding putative laccase is significantly reduced and three genes are significantly increased in expression in the mutant compared with the wild-type Guy11 (Figure 7C). These results indicated that the reduced laccase activity is due to defect in secretory transport.

### MoVam7 plays a role in pathogenicity

**Figure 8:**
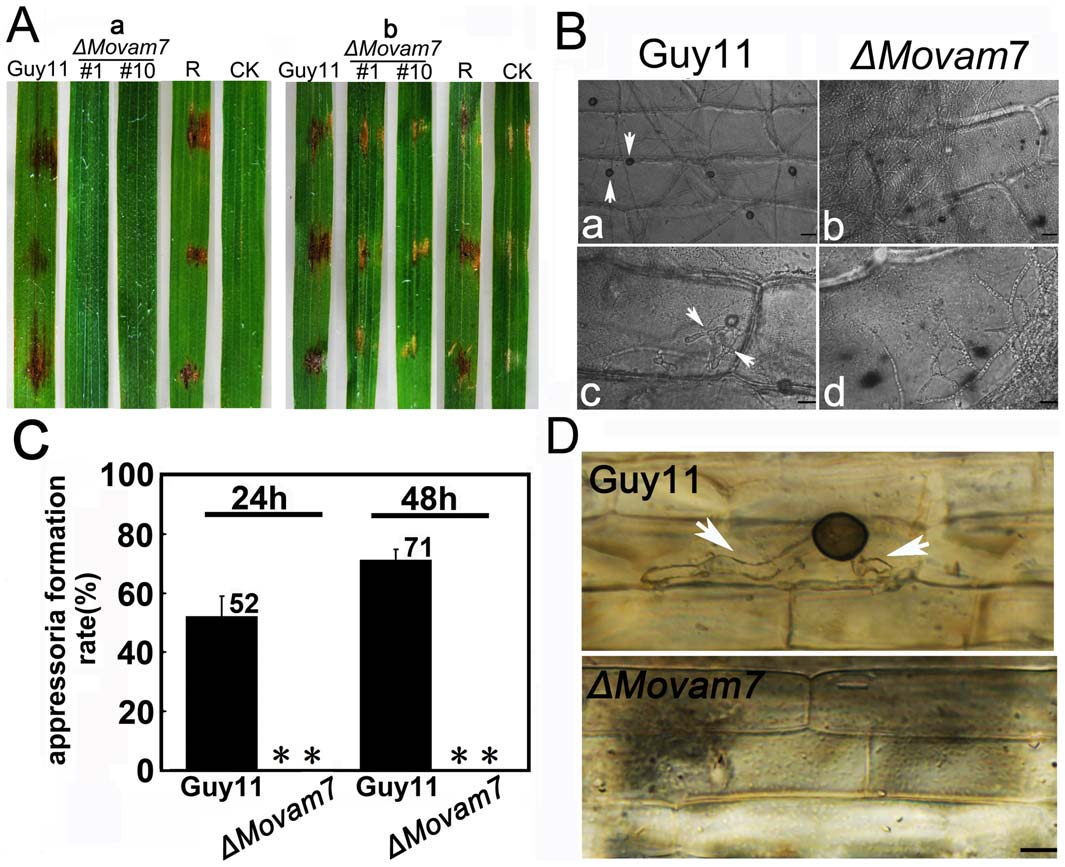
MoVam7 is required for pathogenicity. (A) Pathogenicity test on rice (*Oryza sativa* cv. CO39). Rice leaves, unwounded (a) or wounded by abrasion (b), were inoculated with the wild-type strain, Δ*Movam7* mutants, and complemented mutant (Δ*Movam7*R) strains, with water as a control. Mycelia cultured in CM for 2 days were harvested by centrifugation (10 min at 5000×g), washed twice with distilled water, and fragmented into 30-50 µm lengths by homogenization. The fragmented mycelia suspension was adjusted to 5×10^4^ pieces/ml and inoculated onto intact or abraded leaves of susceptible rice. The experiments were repeated three times each with similar results. (B) Appressorium formation at the hyphal tip was blocked in Δ*Movam7* mutants. The fragmented mycelia suspension was incubated on the surface of hydrophobic Gelbond film as described in Materials and Methods. The appressoria formation rates were obtained at 12 and 24 hours post incubation. (C) and (D) Penetration assays on onion and rice sheath epidermal cells. Fragmented mycelia suspensions (5×10^4^ pieces/ml) of the wild-type and Δ*Movam7* mutant strains were inoculated on strips of onion epidermis and rice sheath, appressoria onion (D) and infectious hyphae on rice sheath (E), all pointed by arrows, were photographed 1 day after inoculation.

Since we were not able to collect enough spores from the Δ*Movam7* mutant for traditional assessment of pathogenicity, we collected mycelia and fragmented them before inoculation on the leaves of susceptible rice cultivar CO-39. The Δ*Movam7* mutant caused no symptoms after 3–5 days, in contrast to the wild-type strain. The same result was obtained when the fragmented mycelia were sprayed on to abraded leaves, demonstrating that MoVam7 is essential for pathogenicity (Figure 8A).

In *M. oryzae*, the cell wall undergoes considerable changes during morphogenesis such as during the formation of appressoria [13]. To test if the Δ*Movam7* mutants was able to form functional appressoria, appressorium assay was performed and results showed that the Δ*Movam7* mutant was unable to form appressoria on the host leaves, whereas plenty of appressoria were found in 52 and 71% of the hyphal tips of the control at 24 and 48 hours post infection (Figure 8C). To test if the loss of the appressoria leads to the loss of tissue colonization, invasive growth and penetration were monitored using the infection model of onion epidermis and rice sheath. Again, the Δ*Movam7* mutants failed to form appressoria and were unable to penetrate the epidermal tissue, whereas the hyphae of the wild type strain were able to form appressoria and develop invasive hyphae underneath the appressoria (Figure 8B and 8D). These results collectively demonstrated that MoVam7 is required for appressorium formation and the infection of the host plant.

## Discussion

Our studies here together the characterization of MoSec22 we reported previously [18] indicated vesicular transport processes mediated by the SNAREs protein play a critical role in the growth, development and pathogenesis of *M. oryzae*.

The Vam7 proteins, as well as many other membrane proteins involved in membrane trafficking, contain a PX domain that targets membranes [40]. MoVam7 is conserved in the regard that its PX domain contains an arginine residue, which specifies its binding to ptdIns(3)P rather than ptdIns(4)P. The implication here is that MoVam7 might preferably target the compartmental ptdIns(3)P containing membranes such as endosomes and vacuoles. Disruption of MoVam7 compromised morphogenesis resulting in many small and fragmented, perhaps immature, vacuoles and endosomes, which might underlie the many other cellular function of MoVam7.

The *Movam7* mutant exhibited a reduction in both growth and the production of conidia, as well as a weakened cell wall and membrane. In *M. oryzae*, the cell wall undergoes considerable changes during morphogenesis [13]. Previous studies have revealed that most cell wall defective mutants with breached cell wall integrity exhibited various developmental defects and were nonpathogenic [41], [42]. It has also been speculated that the polarized growth of filamentous fungi requires the endocytic uptake and recycling of cell wall components such as the chitin [43]. Consistent with these studies, we found that the weakened cell walls and membranes were coupled with abnormal distribution of chitins in the Δ*Movam7* mutants. Thus, disruption of normal membrane trafficking by *MoVAM7* disruption may negatively affect the secretory transport of chitosomes containing chitins and the enzymes that synthesize the chitin. Interestingly, the chitin synthesis related gene appears to be down-regulated in the Δ*Movam7* mutant, in contrast to the Δ*Mosec22* mutant where the chitin synthesis related gene appeared to be up-regulated [18]. How the differentiated expression of these genes regulated by these two SNAREs proteins remains unknown at present time.

During hyphal growth and appressorium formation, endocytic markers were actively internalized by both conidia and conidial germlings on rice leaf, suggesting that endocytosis might play a significant role in spore germination and growth of the germ tube [44]. The FM4-64 staining revealed that endocytosis was blocked by disruption of *MoVAM7*, and the Δ*Movam7* mutants also failed to display the presence of the Spitzenkörper body, the structure associated with fast growing hyphal tips in filamentous fungi. This indicates that growth and differentiation were compromised in the Δ*Movam7* mutants and the mutants would likely be non-pathogenic on host plants. Moreover, previous findings indicated that the accumulation of ROS levels is critical for the development of infection related morphogenesis [37]. Disruption of *MoVAM7* negatively affected ROS levels. One of the possibilities is that MoVam7 is required for the recruitment of ROS generating enzymes to their proper destination, or for the endocytic recycling of plasma membrane carrying the enzymes that synthesize ROS, such as the NADPH oxidase. Conversely, the low ROS levels in the Δ*Movam7* mutants may also be due to a block for targeting the cytosol ROS into proper vacuoles. Nevertheless, the lower ROS levels may be partially contributed to the many defects of Δ*Movam7* including hyphal growth and conidia production.

*M. oryzae* causes rice blast disease that spreads mainly by the asexual spores, which can be transferred from infected plants onto uninfected plants by wind or rain splash dispersal. Both the Δ*Movam7* mutant and the previous reported Δ*Mosec22* mutant are defective in conidiogenesis, appressorium formation and fail to colonize on the plant tissue. Further microscope observation showed that the Δ*Movam7* mutant was able to form conidiophores but most of them failed to differentiate forming spores. This is distinct from the Δ*Mosec22* mutant which was not be able to form conidiophores, suggesting the requirement of vesicular trafficking mediated by different SNAREs is dependent on stages of conidiogenesis.

MoSec22 is important in development and pathogenesis of *M oryzae* [18]. As the results presented here, MoVam7 have distinct as well as redundant roles with MoSec22 in regulated fungal growth, development, and virulence. Both of MoVam7 and MoSec22 have a role in regulating the cell wall integrity, spores formation, accumulation of ROS *in vivo,* and the exocytotic transport of extracellular enzymes, such as extracellular peroxidases and laccases. While both MoSec22 mutant and MoVam7 displayed endocytosis defect, they may function in different pathways. Vam7p in yeast plays its role in vacuole formation and assembly, while the Vam7-like protein Yup1 of *U. maydis* is reported to be critical for endocytosis [3]. Interestingly, in *Aspergillus oryzae*, the AoVam7 was identified to be located at both vacuole and endosome, suggesting AoVam7 is directly involved in endocytosis. As Sec22 of *S. cerevisiae* functions in both anterograde and retrograde traffic between the endoplasmic reticulum (ER) and Golgi apparatus, disruption of MoSec22 may also affect the vesicular trafficking between the ER and Golgi apparatus, comprising the transporting processes from the trans-Golgi network to the PM and affecting the processes of exocytosis and endocytosis. Taken together, our studies suggest that SNARE proteins involved in membrane trafficking are important attributes of fungal virulence and studies of which could potentially lead to new means for the control and management of crop diseases.

## Supporting Information

Table S1 **PCR primers used in this study.**
(DOC)

Figure S1 **Targeted replacement of** ***Magnaporthe oryzae MoVAM7*** **gene.**
(DOC)

Figure S2 **Growth assessment.**
(DOC)

Figure S3 **The effect of MoVam7 on protoplast release by lying enzymes.**
(DOC)

Figure S4 **The role of MoVam7 in chitin contents.**
(DOC)

Figure S5 **Determination of** ***in vivo*** **H_2_O_2_.**
(DOC)
