# Supplementary material for: MoVam7, a Conserved SNARE Involved in Vacuole Assembly, Is Required for Growth, Endocytosis, ROS Accumulation, and Pathogenesis of Magnaporthe oryzae
Source: PLoS One. 2011 Jan 24;6(1):e16439. doi: 10.1371/journal.pone.0016439 (PMC3025985; doi:10.1371/journal.pone.0016439)
Supplement: Table S1 — PCR primers used in this study. (DOC) [file pone.0016439.s001.doc]

**Table S1.** PCR primers used in this study.

| Primer No. | Oligonucleotide sequence | Gene ID |
| --- | --- | --- |
| FL474 | 5'-TCGACGTCCGAAAGGATCTGT-3' | MGG_03982 |
| FL475 | 5'-ACTCCTGCTTCGAGATCCACATC-3' |
| FL724 | 5'-TGACTGGGTGGGTGTATTCAGAGAA-3' |  |
| FL725 | 5'-GCTGCTTGACCTCCTCCTCGATCgatatcCGGAGTAGCGCTTCTGCACGACA-3' |  |
| FL726 | 5'-TGTCGTGCAGAAGCGCTACTCCGgatatcGATCGAGGAGGAGGTCAAGCAGC-3' |  |
| FL727 | 5'-GCCGCTGTGATTCAACTTGTGACTCA-3' |  |
| FL1111 | 5'-GGAGGTCAACACATCAATG-3' |  |
| FL1112 | 5'-CTCTATTCCTTTGCCCTCG-3' |  |
| FL1464 | 5'-CGCaagcttAGTTCAACGTCAACAGCGAATATG-3' |  |
| FL1465 | 5'-CCTggatccCTGTTGCCACGACAACCGATATTG-3' |  |
| FL1466 | 5'-ATGCCACCACCAGTCGAGATCTCG-3' | MGG_05428 |
| FL1467 | 5'-TCACGACCCCAGCTTCCTCGTCCG-3' |
| FL2194 | 5'-GTCCAGCTGGAGGAGTACCTACA-3' |
| FL2195 | 5'-CTGCGTCAGCCACCTGTGGTCCT-3' |
| FL4362 | 5'-CCATGTACCCTGGTCTTTCG-3' | MGG_03982 |
| FL4363 | 5'-TTCGAGATCCACATCTGCTG-3' |
| FL4394 | 5'-ACATGGACACCACCCAGAAC-3' | MGG_00750 |
| FL4395 | 5'-CATGCTTCCCTCGAGACCAC-3' |
| FL4396 | 5'-ATCAAGGCCGATGATGCTAC-3' | MGG_06559 |
| FL4397 | 5'-ATGTGCTACCCAGACCCTTG-3' |
| FL4712 | 5'-ACGCCGTCTACTCAGGATCA-3' | MGG_02252 |
| FL4713 | 5'-TCTCGCCGTTTGGAATGTAT-3' |
| FL4781 | 5'-TACTTTGACGACGCCATGAC-3' | MGG_08200 |
| FL4782 | 5'-CCCGACGGTATGTGCTAAGT-3' |
| FL4787 | 5'-CTGCTGCTCAACGAGAAGTG-3' | MGG_04545 |
| FL4788 | 5'-ACACCGAGCTCAAACAGCTT-3' |
| FL4799 | 5'-CCACGAGCTCAACTTTGGAT-3' | MGG_04404 |
| FL4800 | 5'-GGACGGTGACAAGCATCTCT-3' |
| FL4803 | 5'-TCCCTGGACTGCTCAAGTCT-3' | MGG_01924 |
| FL4804 | 5'-GCTGTCAAGAGGACGGTAGC-3' |
| FL4807 | 5'-ACAACGGTCGCCTTTTACAC-3' | MGG_02069 |
| FL4808 | 5'-GTGTTGGTGTTGGCCTTTTC-3' |
| FL4929 | 5'-TGCTGCTCATGTCCACCTAC-3' | MGG_01802 |
| FL4930 | 5'-TCCTCTTGAGGCTTGTCGAT-3' |
| FL4931 | 5'-CTTGTTGCAAAGCGAGATGA-3' | MGG_04145 |
| FL4932 | 5'-CCTGGAGAAGCTGGTAGACG-3' |
| FL4933 | 5'-CGACCAGCTTCAACTTCACA-3' | MGG_09551 |
| FL4934 | 5'-GGAGTCTGAGCTTCGTTTGG-3' |
| FL4935 | 5'-AAACTCGAGGGACATGTTGG-3' | MGG_06064 |
| FL4936 | 5'-CCTCCTGAACGCAGAGAAAC-3' |
| FL4937 | 5'-TCCTGATGTCGTTCTTGCAG-3' | MGG_09962 |
| FL4938 | 5'-GATCTCAGGGTCCTTCACCA-3' |
| FL4939 | 5'-TATGCGCTACGATGACAAGC-3' | MGG_13013 |
| FL4940 | 5'- CGAGTAAACCTTGCCCATGT-3' |
| FL4941 | 5'-CACCTGCGTTTACCTTGGAT-3' | MGG_13014 |
| FL4942 | 5'-TACCCCACGAGAAGTTGTCC-3' |
| FL5029 | 5'-CTCGCTTGACAACCACACC-3' | MGG_05790 |
| FL5030 | 5'-CCGAGGCTTGACCGTAGTAG-3' |
| FL5031 | 5'-CAGTACATCGCGGAGAAAAA-3' | MGG_08127 |
| FL5032 | 5'-GACGGGGTTATTGAAGTTGAG-3' |
| FL5035 | 5'-GTCAACAACGCTGCTCTCC-3' | MGG_13764 |
| FL5036 | 5'-GGGGTCCGTGATGATGTAG-3' |
| FL5037 | 5'-TTACAACGACCCCAACTTCAT-3' | MGG_08046 |
| FL5038 | 5'-CCTTTCCCGCTGTCATTCT-3' |
| FL4795 | 5'-GGAGTACTGGCTCTCCATCG-3' | MGG_11608 |
| FL4796 | 5'-TTGGTCTGCATGTTGTTGGT-3' |
|  |  |  |
|  |  |
|  |  |  |
|  |  |
|  |  |  |
|  |  |
|  |  |  |
|  |  |
