# Supplementary material for: MoVam7, a Conserved SNARE Involved in Vacuole Assembly, Is Required for Growth, Endocytosis, ROS Accumulation, and Pathogenesis of Magnaporthe oryzae
Source: PLoS One. 2011 Jan 24;6(1):e16439. doi: 10.1371/journal.pone.0016439 (PMC3025985; doi:10.1371/journal.pone.0016439)
Supplement: Figure S1 — Targeted replacement of Magnaporthe oryzae MoVAM7 gene. (DOC) [file pone.0016439.s002.doc]

**Figure S1.**  Targeted replacement of the *Magnaporthe oryzae MoVAM7* gene.


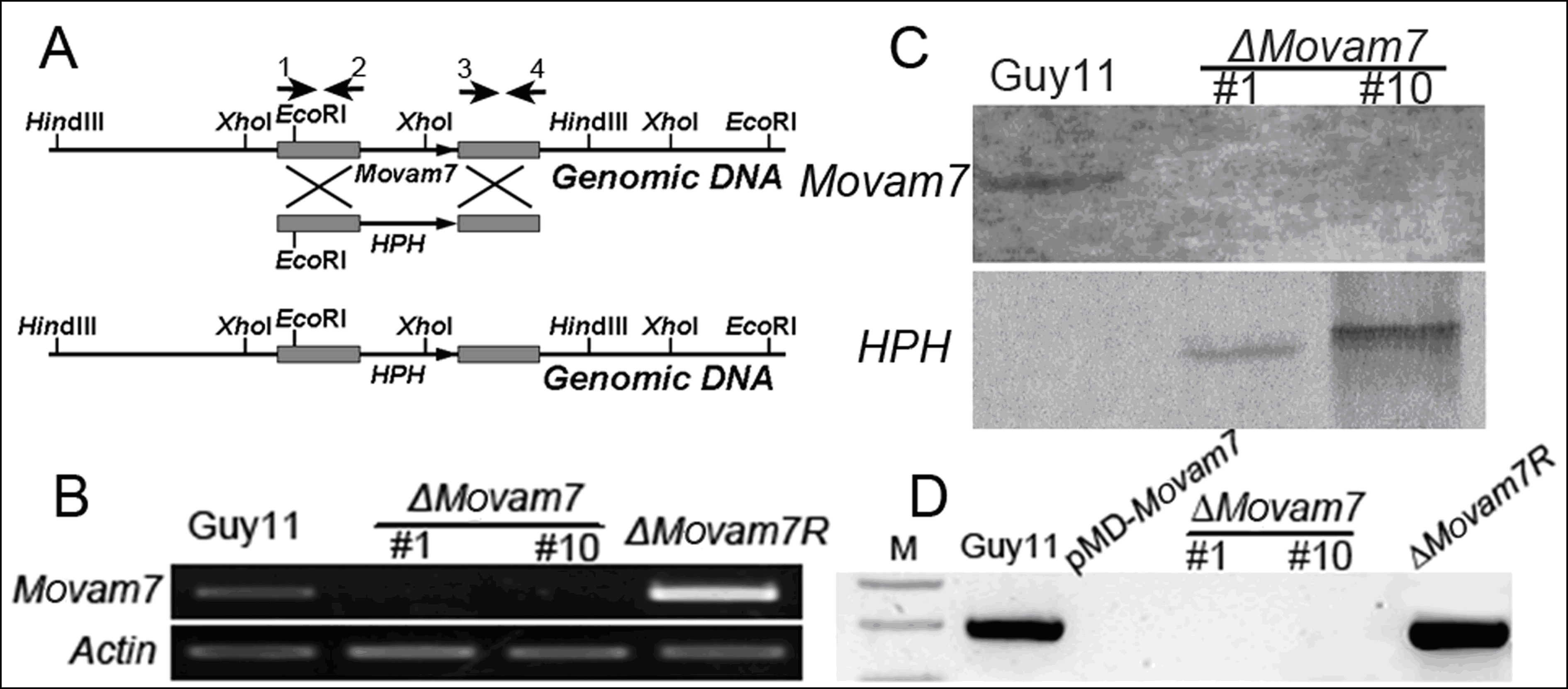


(A) Schematic illustration for *MoVAM7* targeted gene replacement. Positions and orientations of primers FL724 (1), FL725 (2), FL726 (3), and FL727 (4) are labeled with small arrows. The *Movam7::HPH* deletion allele was amplified with primers FL724/FL727, purified by gel electrophoresis, and used to transform the protoplasts of *M. oryzae* wild type strain. (B) ∆*Movam7* mutants were verified by RT-PCR with *MoVAM7* specific primers the actin gene as a internal control. (C) ∆*Movam7* mutants were verified by Southern blotting analysis. Genomic DNA was digested with *Hind*III and separated in a 0.7% agarose gel. The DNA was hybridized with probe A amplified by primers FL2194/FL2195 and probe B amplified by primers FL1111/FL1112 for a 1.4 kb *HPH* fragment. (D) ∆*Movam7* mutants were verified by PCR. Transformants #1 and #10 were representative mutants. The *Movam7*R were obtained by re-introduction of the wild-type *MoVAM7* gene into the ∆*Movam7* mutant.
