# Supplementary material for: MoVam7, a Conserved SNARE Involved in Vacuole Assembly, Is Required for Growth, Endocytosis, ROS Accumulation, and Pathogenesis of Magnaporthe oryzae
Source: PLoS One. 2011 Jan 24;6(1):e16439. doi: 10.1371/journal.pone.0016439 (PMC3025985; doi:10.1371/journal.pone.0016439)
Supplement: Figure S2 — Growth assessment. (DOC) [file pone.0016439.s003.doc]

**Figure S2.** Growth assessment.


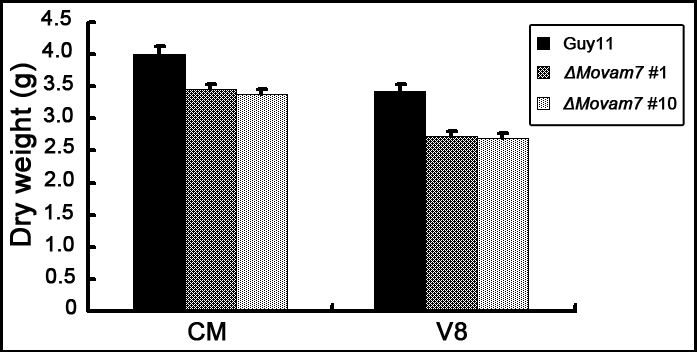


Fungal cultures grown in CM and V8 liquid media were processed for dry weight measurement. Data comprise three independent experiments with triple replications each time that yielded similar results.
