# Supplementary material for: MoVam7, a Conserved SNARE Involved in Vacuole Assembly, Is Required for Growth, Endocytosis, ROS Accumulation, and Pathogenesis of Magnaporthe oryzae
Source: PLoS One. 2011 Jan 24;6(1):e16439. doi: 10.1371/journal.pone.0016439 (PMC3025985; doi:10.1371/journal.pone.0016439)
Supplement: Figure S3 — The effect of MoVam7 on protoplast release by lying enzymes. (DOC) [file pone.0016439.s004.doc]

**Figure S3.** The effect of MoVam7 on protoplast release by lying enzymes.


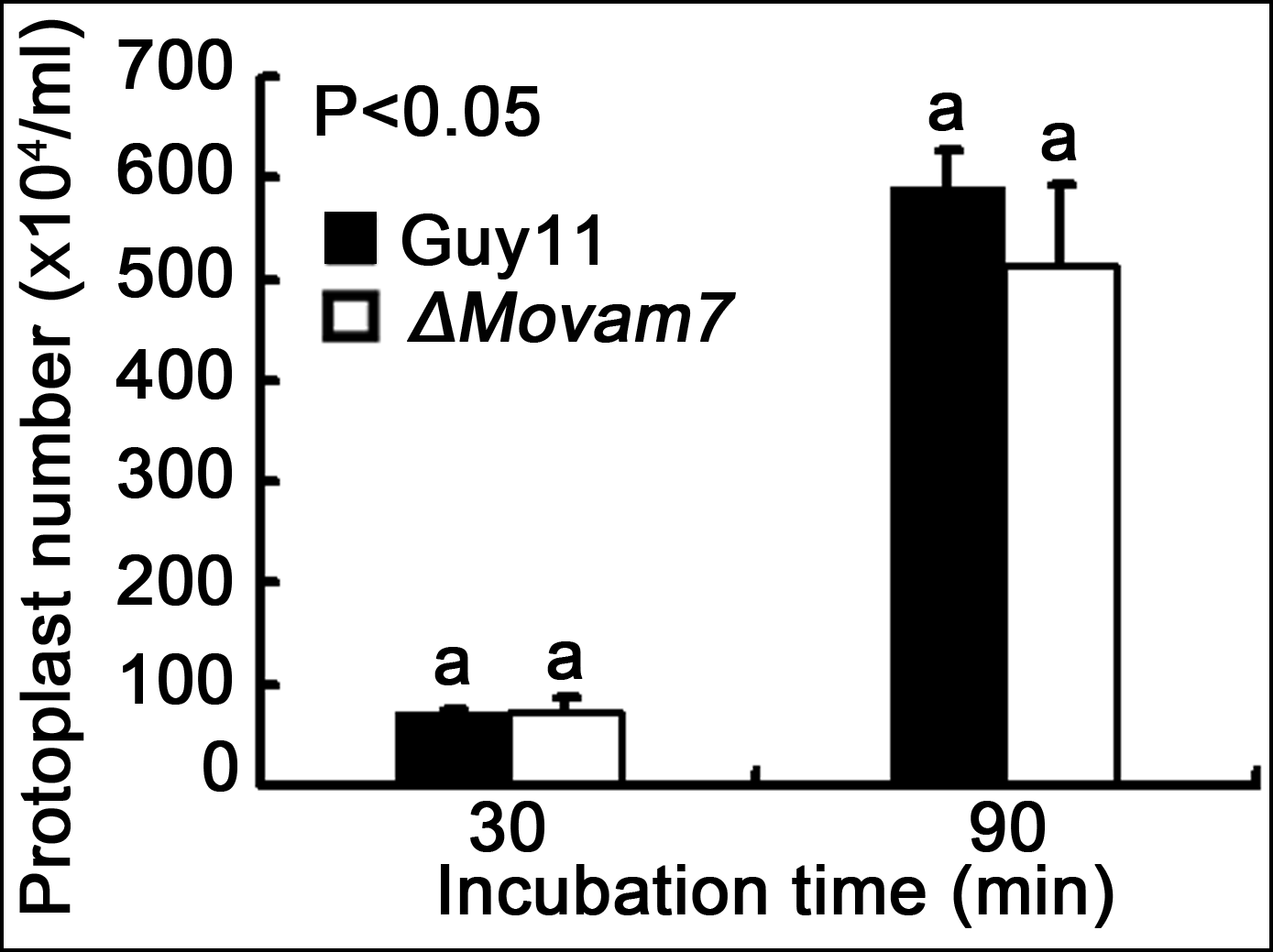


No significant difference were observed in protoplast release by lytic enzymes between the ∆*Movam7* mutant and wild-type strains at *p* = 0.05, according to Duncan’s range test.
