# Supplementary material for: MoVam7, a Conserved SNARE Involved in Vacuole Assembly, Is Required for Growth, Endocytosis, ROS Accumulation, and Pathogenesis of Magnaporthe oryzae
Source: PLoS One. 2011 Jan 24;6(1):e16439. doi: 10.1371/journal.pone.0016439 (PMC3025985; doi:10.1371/journal.pone.0016439)
Supplement: Figure S4 — The role of MoVam7 in chitin contents. (DOC) [file pone.0016439.s005.doc]

**Figure S4.** The role of MoVam7 in chitin contents

**
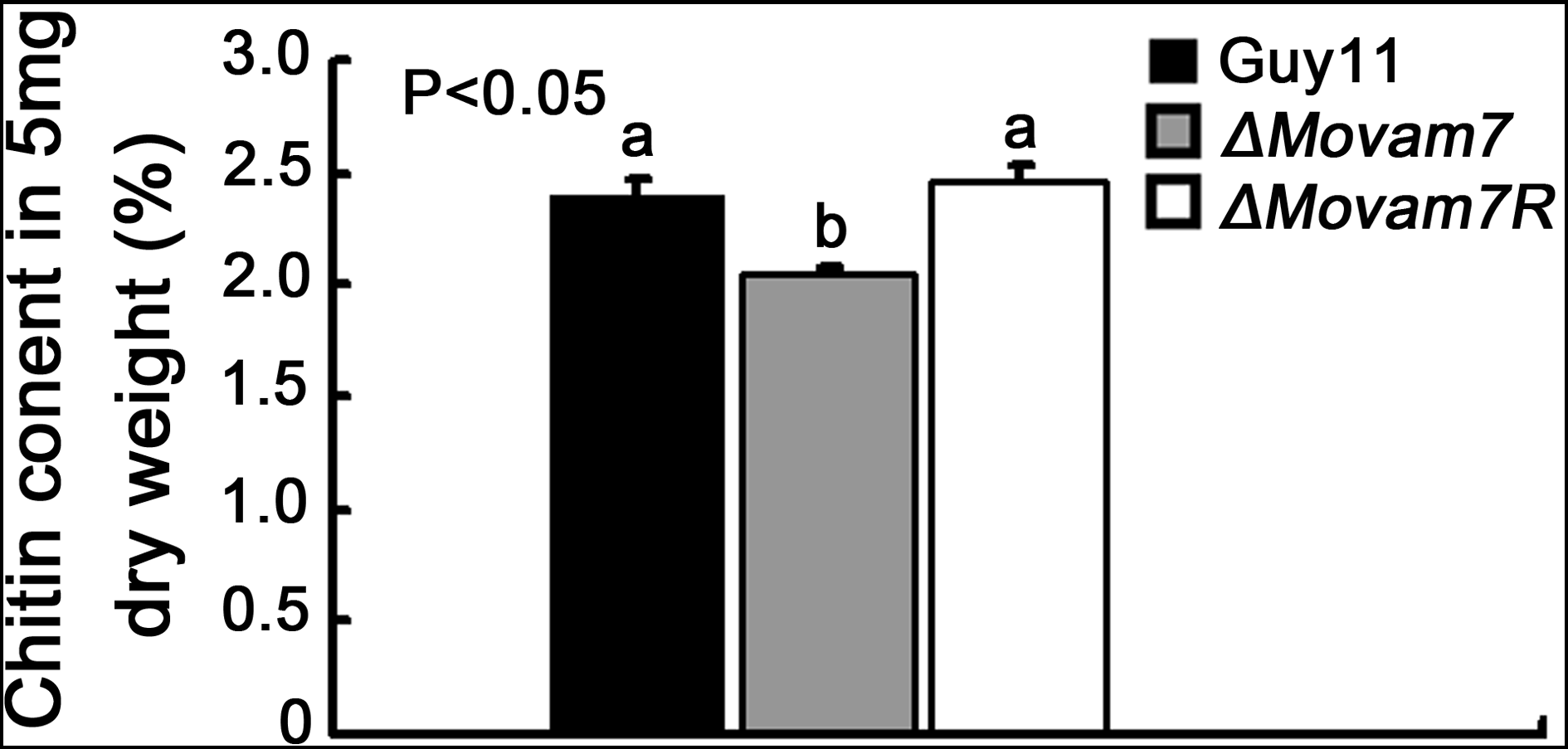
**

GlcNa determination by the fluorimetric Morgan–Elson method shows reduced chitin contents in the mutant compared to the wild-type strain and the reconstituted strain. Different capital letter indicate a significant difference between the chitin content in the mutant and wild-type strain (or the reconstituted strain) at *p* = 0.05, according to Duncan’s range test. Data comprise three independent experiments with triple replications each time that yielded similar results.
