# Supplementary material for: MoVam7, a Conserved SNARE Involved in Vacuole Assembly, Is Required for Growth, Endocytosis, ROS Accumulation, and Pathogenesis of Magnaporthe oryzae
Source: PLoS One. 2011 Jan 24;6(1):e16439. doi: 10.1371/journal.pone.0016439 (PMC3025985; doi:10.1371/journal.pone.0016439)
Supplement: Figure S5 — Determination of in vivo H2O2. (DOC) [file pone.0016439.s006.doc]

**Figure S5.** *in vivo* determination of H_2_O_2_

**
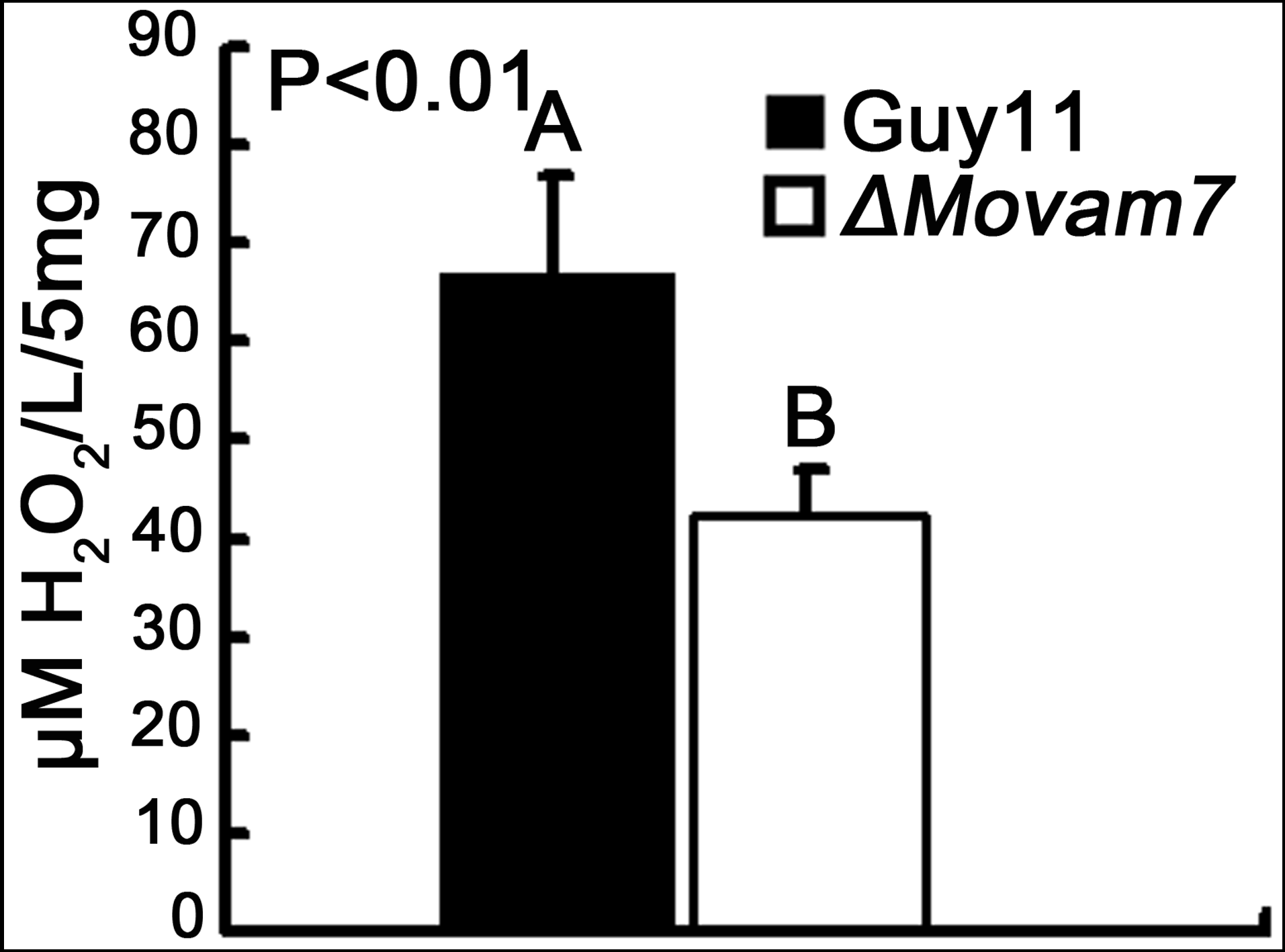
**

Different capital letter indicate a significant difference between the H_2_O_2_ content in the mutant and wild-type strains at *p* = 0.01, according to Duncan’s range test. Data comprise three independent experiments with triple replications each time that yielded similar results.
